# The *pip1s* Quintuple Mutants Demonstrate the Essential Roles of *PIP1s* in the Plant Growth and Development of *Arabidopsis*

**DOI:** 10.3390/ijms22041669

**Published:** 2021-02-07

**Authors:** Xing Wang, Yu Wu, Zijin Liu, Tong Liu, Lamei Zheng, Genfa Zhang

**Affiliations:** Beijing Key Laboratory of Gene Resource and Molecular Development, College of Life Sciences, Beijing Normal University, Beijing 100875, China; xingwang163@163.com (X.W.); wuyu_0122@163.com (Y.W.); liuzijin1012@mail.bnu.edu.cn (Z.L.); 201721200025@mail.bnu.edu.cn (T.L.); zhenglm100@163.com (L.Z.)

**Keywords:** *Arabidopsis*, PIP1s, vegetative growth, reproductive growth, transcriptome

## Abstract

Plasma membrane intrinsic proteins (PIPs) transport water, CO_2_ and small neutral solutes across the plasma membranes. In this study, we used the clustered regularly interspaced short palindromic repeats (CRISPR) and CRISPR-associated protein 9 system (CRISPR/Cas9) to mutate *PIP1;4* and *PIP1;5* in a *pip1;1,2,3* triple mutant to generate a *pip1;1,2,3,4,5* (*pip1s^−^*) quintuple mutant. Compared to the wild-type (WT) plant, the *pip1s*^−^ mutants had smaller sized rosette leaves and flowers, less rosette leaf number, more undeveloped siliques, shorter silique and less seeds. The pollen germination rate of the *pip1s^−^* mutant was significantly lower than that of the WT and the outer wall of the *pip1s^−^* mutant’s pollen was deformed. The transcriptomic analysis showed significant alterations in the expression of many key genes and transcription factors (TFs) in the *pip1s*^−^ mutant which involved in the development of leaf, flower and pollen, suggesting that the mutant of *PIP1s* not only directly affects hydraulics and carbon fixation, but also regulates the expression of related genes to affect plant growth and development.

## 1. Introduction

Water movement is a key physiological process of vegetative and reproductive growth in plants that must be tightly regulated under different conditions [1,2,3,4,5]. The transport of water is controlled by both symplastic and apoplastic pathways. Aquaporins (AQPs) play a central role in the symplastic pathway which is efficient in transporting water across membranes [6,7,8]. Aquaporins transport water, CO_2_ and small neutral solutes through the plasma membranes and intracellular membranes of cells in many physiological and developmental processes, including cell elongation, stomatal regulation, seed germination, reproductive growth and stress responses in plants [1,5,7,8].

According to the conserved amino acid sequences and intron positions, AQPs are divided into five groups including plasma membrane intrinsic proteins (PIPs), tonoplast intrinsic proteins (TIPs), nodulin-like plasma membrane intrinsic proteins (NIPs), small intrinsic proteins (SIPs), and X intrinsic proteins (XIPs) [9,10,11,12]. PIPs are located to the plasma membrane and regulate water movement between cells to maintain the water balance [7,13]. Sequences of the *PIP1s* and *PIP2s* subgroups are highly conserved, however, the two isoforms are different in many aspects including their transport properties and subcellular localization.

The PIP2s subgroup is an efficient water channel, but the water transport capacity of PIP1s is inconclusive. Although some PIP1 has been proven to be an effective water channel in some species [14,15,16], the water transport efficiency of other PIP1s is relatively low or even none [17,18,19,20]. Subsequent studies have shown that this may be due to singly expressed PIP1s are retained intracellularly and fail to traffic to the plasma membrane [21,22]. Co-expression studies in Xenopus oocytes or maize protoplasts have clearly elucidated that PIP2s physically interact with PIP1s to form a heterotetramer [21,22]. PIP2s promote the traffic of PIP1s from the endoplasmic reticulum (ER) to the plasma membrane and enhance the water transport efficiency of PIP1s [23]. Interestingly, studies in maize showed that the water transport efficiency of ZmPIP1;2 and ZmPIP2;5 heterotetramer was even higher than that of ZMPIP2;5 homotetramer [24]. The role of PIP1s as modulators of membrane water permeability has also been reported in garden strawberry [25].

According to previous studies, the transcriptional expression, biological activity and localization of PIP1s and PIP2s are regulated by abiotic stresses, plant hormones and light [2,3,4,5,26,27]. Overexpression of *PIPs* in plants may lead to more stress resistant or less, which depend on the sources or isoforms of these genes [28,29,30,31,32,33,34,35,36]. Therefore, specific *PIPs*, which confer plants more resistance to stresses or better growth and development, are important potential genetic resources in agronomic and crop science [16,19,37,38,39,40,41,42,43,44].

We constructed single mutants, double mutants and triple mutants to study the functions of PIP1s. Our work found that under natural growth conditions, the phenotypes of rosette leaves, flowers and siliques were not significantly altered in these mutants. However, under drought and salt stress conditions, the mutants showed a slight difference in seed germination rates and survival rates compared to the WT plant. The quintuple *pip1s*^−^ mutant must be generated to further refine the roles of PIP1s in plant growth and development. However, the mutant is difficult by crossing corresponding T-DNA insert mutants. In this study, we generated quintuple *pip1s*^−^ mutants by mutating PIP1;4 and PIP1;5 in the *pip1;1/2/3* triple mutant using the CRISPR/cas9 system. The study aims to determine the role of the *PIP1s* subgroup in plant growth and development and to provide a theoretical basis of agricultural production to improve crop traits and yield.

## 2. Results

### 2.1. Generation of the pip1s^−^ Quintuple Mutants in Arabidopsis Thaliana

**Figure 1:**
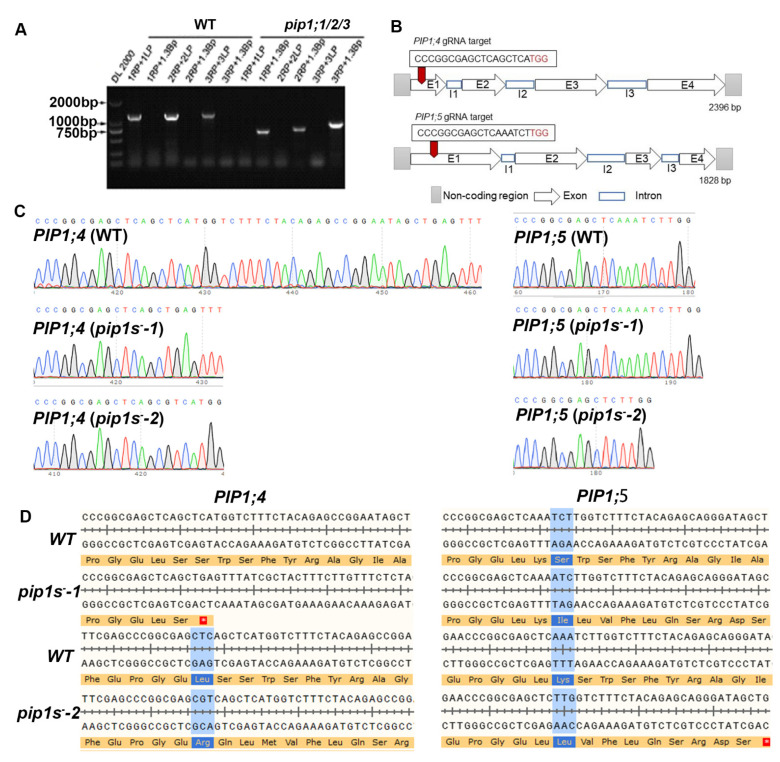
Generation of the *pip1s* mutants. (**A**) Amplification of the T-DNA insertion in mutant *pip1;1/2/3*. (**B**) Schematic of the *pip1s* mutant. (**C**) Sanger sequencing chromatographs showing the deletion in the *pip1s* mutant. (**D**) Amino acid sequence alignment of the *pip1s^−^* mutant and the WT plant. The asterisk on the red background represents the termination codon.

To explore the contributions of *PIP1s* during the vegetative growth and reproductive growth, the CRISPR-cas9 system was used to mutate the *PIP1;4* and *PIP1;5* in the *pip1;1/2/3* triple mutant. PCR showed that full-length of *PIP1;1/2/3* was not expressed in the *pip1;1/2/3* mutant (Figure 1A). The pVK004-15 plasmids expressing the sgRNA targets for *PIP1;4* and *PIP1;5* was transformed into the *pip1;1/2/3* mutant to generate the *pip1s*^−^ quintuple mutant (Figure 1B). The homozygous *pip1s*^−^ quintuple mutant lines among the T_2_ plants were identified by sequencing. Sequencing results identified a 29-bp deletion in the coding region of *PIP1;4* and a 1-bp insertion in the coding region of *PIP1;5* in *pip1s^−^-1* (Figure 1C). A 1-bp insertion in the coding region of *PIP1;4* and a 5-bp deletion in the coding region of *PIP1;5* were detected in *pip1s^−^-2* (Figure 1C). Both mutated *PIP1;4* and *PIP1;5* sequences in *pip1s^−^-1* and *pip1s^−^-2* were frameshift mutations, resulting in the premature translational termination of *PIP1;4* and *PIP1;5* (Figure 1D).

### 2.2. The pip1s^−^ Quintuple Mutants Are Defective in Plant Vegetative Growth

**Figure 2:**
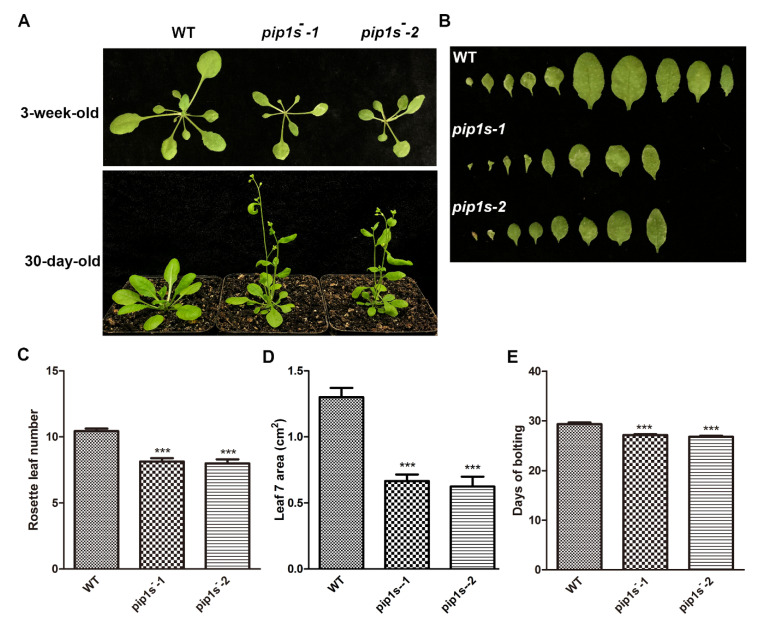
The *pip1s*^−^ mutants are defective in vegetative growth in *Arabidopsis*. (**A**) The growth phenotypes of the WT, *pip1s^−^-1* and *pip1s^−^-2* plants. The 10-day-old seedlings grown on 1/2 MS plates were transplanted to soil and grown at 22 °C with 16 h/8 h light/dark. Photographs were captured 30 days or 3 weeks after transplantation. (**B**) Morphological phenotype of the leaves of 3-week-old WT, *pip1s^−^-1* and *pip1s^−^-2* plants. (**C**) Number of rosette leaves produced per plant of the WT, *pip1s^−^-1* and *pip1s*^−^*-2* plants after transplanted for 3 weeks. (**D**) The area of the seventh rosette leaf of the WT, *pip1s^−^-1* and *pip1s^−^-2* plants after transplanted for 3 weeks. The area was determined with ImageJ software. (**E**) Days to start bolting of the WT, *pip1s*^−^-1 and *pip1s*^−^-2 plants. Bars represent the means ± SD of three biological replicates. Significant differences were determined by one-way analysis of variance (ANOVA) followed by Duncan’s multiple range test (*** *p* < 0.0001).

The WT, *pip1s^−^-1*, and *pip1s^−^-2* plants were grown on soil under 16/8 h light/dark conditions to investigate the phenotypes of the *pip1s^−^*. Compared to the WT plant, the *pip1s^−^* mutants had smaller rosette leaves and fewer rosette leaves during the vegetative stage (Figure 2A–D). Early bolting was observed in the *pip1s*^−^ mutants under normal growth conditions (Figure 2A). The bolting time in the *pip1s*^−^ mutants was significantly advanced (up to 3 days) compared to the WT plants (Figure 2E).

### 2.3. Mutation of PIP1s Affects Flower Growth and Development

**Figure 3:**
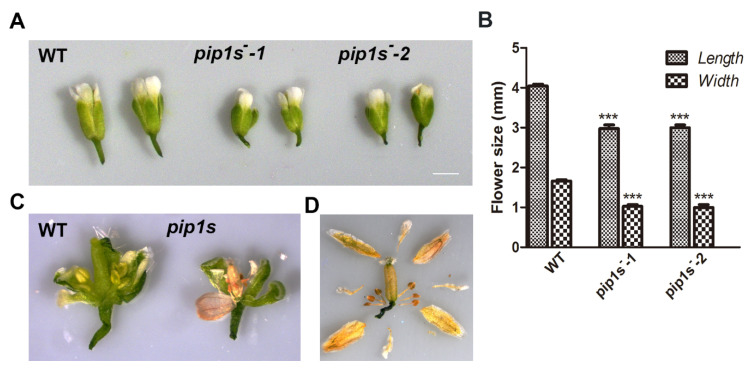
**Analysis of the** flower morphology of the WT, *pip1s^−^-1* and *pip1s^−^-2* plants. (**A**) Photographs of flowers from the 5-week-old plants grown under standard conditions were captured. (**B**) Statistics analysis of flower length and width of the WT, *pip1s^−^-1*, and *pip1s^−^-2* plants. (**C**) Morphology of dissected flowers from the WT and *pip1s*^−^. (**D**) Morphological phenotype of the dissected dry flower buds of *pip1s*^−^ plants. Bars represent the means ± SD of three biological replicates. Significant differences were determined by one-way ANOVA followed by Duncan’s multiple range test (*** *p* < 0.0001).

Compared to the WT plants, significantly reduced lengths and widths of mature flowers were observed in *pip1s^−^-1* and *pip1s^−^-2* mutants (Figure 3A,B). Many flower buds of *pip1s^−^* mutants began to wither before flowering (Figure 3C). Eventually, the sepals, petals, stamens, and all carpels of the flower withered (Figure 3D).

### 2.4. Mutation of PIP1s Decreases the Number and Length of Silique

**Figure 4:**
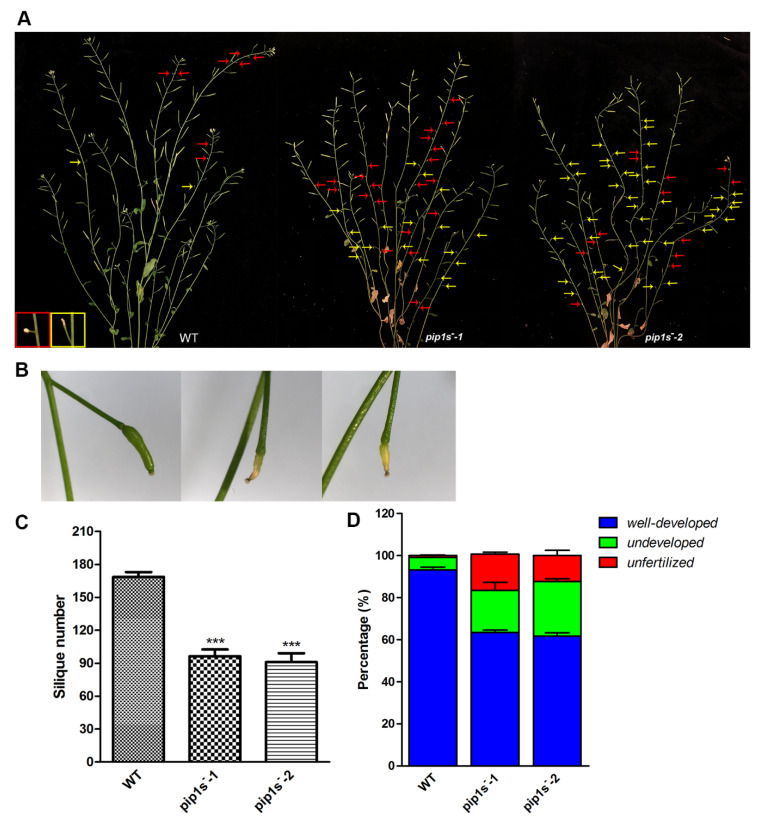
*PIP1s* mutations impair plant fertility at the reproductive stage. (**A**) Phenotypes of the WT, *pip1s^−^-1*, and *pip1s^−^-2* plants during the reproductive stage. Photographs of 8-week-old plants were captured. Red arrows indicate unfertilized flowers; yellow arrows indicate undeveloped siliques. (**B**) Phenotypes of well-developed and undeveloped siliques. (**C**) Statistical analysis of the number of siliques per plant. (**D**) Siliques were divided into three categories and the percentage was calculated. Bars represent the means ± SD of three biological replicates. Significant differences were determined by one-way ANOVA followed by Duncan’s multiple range test (*** *p* < 0.0001).

Unfertilized or withered flowers and undeveloped siliques were observed in both WT and *pip1s*^−^ mutant plants (Figure 4A). Undeveloped siliques turned yellow, wilted, and even moldy during silique development (Figure 4B). The number of siliques per plant was significantly reduced in the *pip1s*^−^ mutants compared to the WT plants (*p* < 0.01) (Figure 4C). We divided these siliques into three major categories: well-developed, undeveloped, and unfertilized (Figure 4D). The percentages of undeveloped and unfertilized siliques were substantially increased in the *pip1s*^−^ mutants.

**Figure 5:**
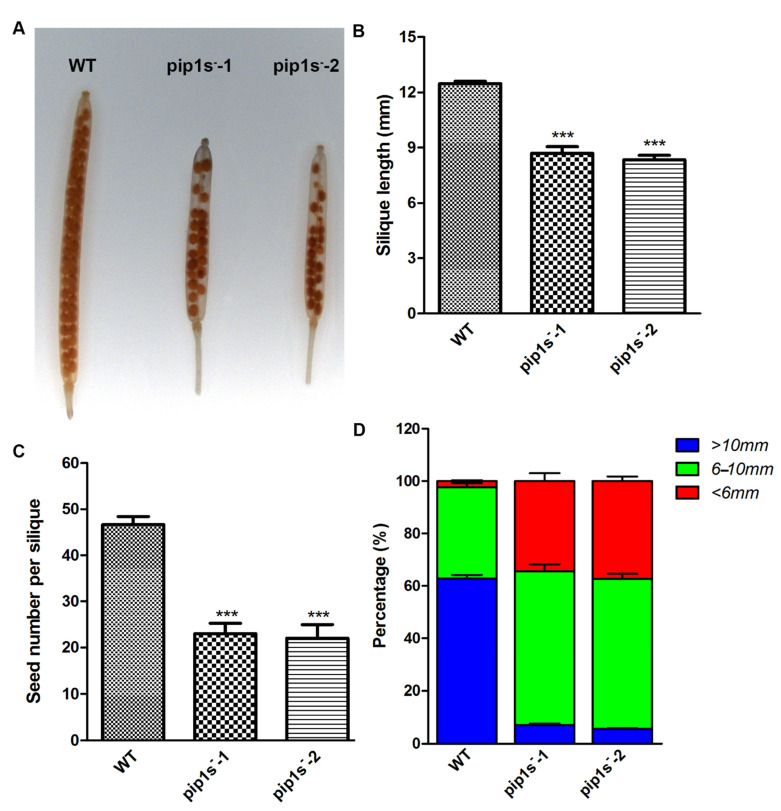
Analysis of the phenotypes of siliques from the WT, *pip1s^−^-1* and *pip1s^−^-2* plants. (**A**) Transparent siliques of the WT, *pip1s^−^-1*, and *pip1s^−^-2* plants at 14 DAF. Bar = 5 mm. (**B**) Length of 14 DAF siliques. (**C**) Statistical analysis of seed number per silique at 14 DAF. (**D**) Silique length was divided into three categories and the percentage per plant was calculated. Bars represent the means ± SD of three biological replicates. Significant differences were determined by one-way ANOVA followed by Duncan’s multiple range test (*** *p* < 0.0001).

When comparing the developed siliques of the WT and *pip1s*^−^ mutant plants, we found that the mutant siliques were nicked (Figure 5A). The *pip1s*^−^ mutants had shorter silique lengths than the WT plants at 14 days after flowering (DAF) (Figure 5B). The average seed number per silique of the WT was 46.7, which was significantly greater than that of the *pip1s^−^-1* (23.8) and *pip1s^−^-2* (23.1) (*p* < 0.01) (Figure 5C). We divided the silique length into three categories: >10 mm, 6–10 mm, and <6 mm. The silique lengths of the *pip1s^−^-1* and *pip1s^−^-2* mutants were largely reduced compared to the WT plants (Figure 5D).

### 2.5. Mutation of PIP1s Affects Seed Development

**Figure 6:**
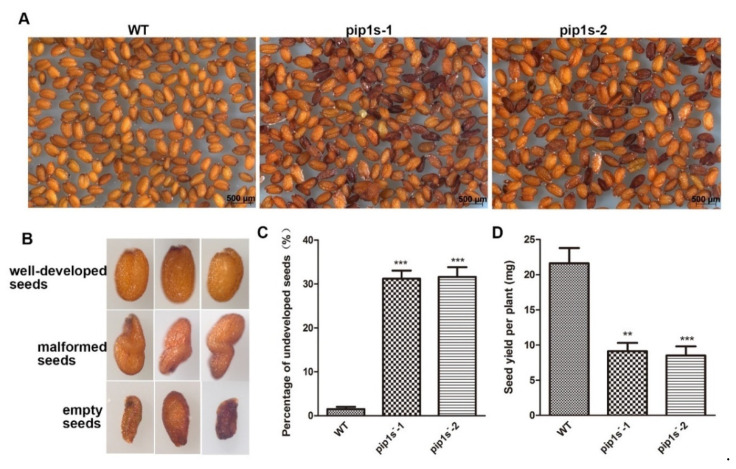
Phenotypic analysis of seeds from the WT, *pip1s^−^-1* and *pip1s^−^-2* plants. (**A**) The seeds of WT, *pip1s*^−^*-1* and *pip1s^−^-2* plants. (**B**) The phenotype of well-developed seeds, malformed seeds and aborted seeds. (**C**) Statistical analysis of the percentage of undeveloped seeds. (**D**) Statistical analysis of the seed yield per plant of the WT, *pip1s^−^-1* and *pip1s^−^-2* plants. Bars represent the means ± SD of three biological replicates. Significant differences were determined by one-way ANOVA followed by Duncan’s multiple range test (** *p* < 0.0005, *** *p* < 0.0001).

Compared to the WT plants, some aborted and malformed seeds were observed in the *pip1s*^−^ mutants (Figure 6A,B). The proportions of the aborted seeds in the *pip1s^−^-1* and *pip1s^−^-2* mutants were 31.2% and 31.6%, respectively (Figure 6C).

### 2.6. Mutation of PIP1s Affects Pollen Morphology and Activity

**Figure 7:**
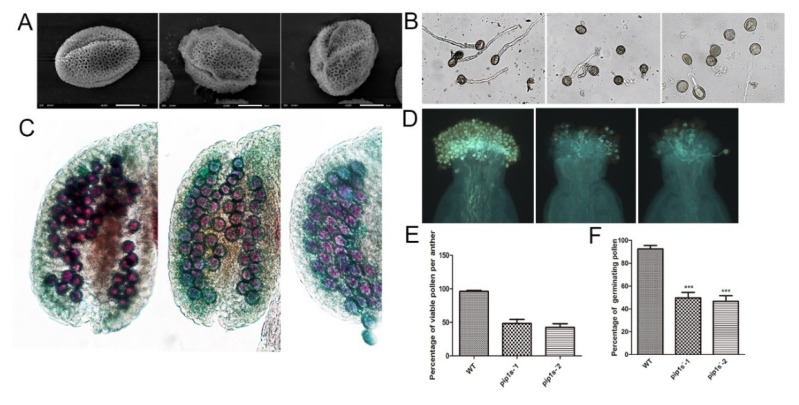
Pollen morphology and vitality of the WT, *pip1s^−^-1* and *pip1s^−^-2* plants. (**A**) Scanning electron micrographs of pollen from the WT, *pip1s^−^-1*, and *pip1s^−^-2* plants. (**B**) Pollen germination in liquid culture media in vitro. (**C**) Alexander-stained anthers of WT, *pip1s^−^-1*, and *pip1s^−^-2* plants. (**D**) Aniline blue stained pollen and pollen tubes of WT, *pip1s*^−^*-1*, and *pip1s^−^-2* plants. (**E**) Statistical analysis of the percentage of viable pollen grains per anther. (**F**) Statistical analysis of the percentages of germinating pollen and undeveloped seeds. Bars represent the means ± SD of three biological replicates. Significant differences were determined by one-way ANOVA followed by Duncan’s multiple range test (*** *p* < 0.0001).

Scanning electron microscopy indicated that both the WT and *pip1s*^−^ mutant pollen had regular honeycomb outer wall structures. However, the outer wall of the mutant pollen was deformed and shrunk (Figure 7A). Pollen germination experiments conducted in vitro revealed that the pollen germination rate of the *pip1s*^−^ mutant was significantly lower than the WT plant (*p* < 0.01) (Figure 7B,F). The pollen germination experiment conducted in vivo produced similar results, and the pollen-stigma binding ability of the *pip1s*^−^ mutant was impaired compared to the WT plant (Figure 7D). Alexander staining experiments displayed a lower number of active pollen grains per anther in the *pip1s*^−^ mutant than in the WT plant (Figure 7C).

### 2.7. Analysis of Differentially Expressed Genes (DEGs) in the pip1s^−^ Mutants and the WT Plants

**Figure 8:**
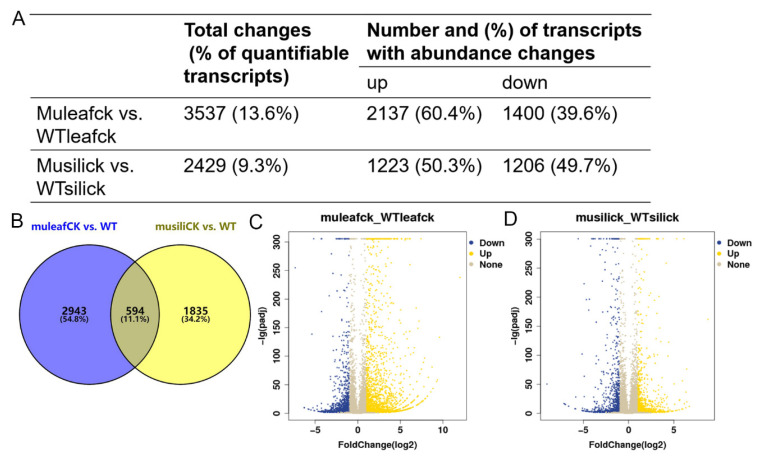
Transcriptome analysis of the WT and *pip1s*^−^ mutant plants. (**A**) The number of DEGs in terms of transcript abundance in rosette leaves and siliques from the WT and *pip1s*^−^ mutant plants. (**B**) Venn diagram showing the number of DEGs in the two comparison groups (muleaf vs. WT and musili vs. WT). (**C**,**D**) Volcano plots showing up-regulated and down-regulated of DEGs in the comparisons of muleaf with WTleaf (**C**) and musili with WTsili (**D**).

To investigate the effect of *PIP1s* on the transcriptome profile, 30-day-old rosette leaves and 14 DAF siliques were collected from the WT and *pip1s*^−^ mutant plants for the RNA-seq analysis. At the transcriptional level, the expression of 3537 genes was significantly changed (|log_2_| ≥ 1, FDR ≤ 0.05) in the *pip1s*^−^ mutant rosette leaves compared to the WT (muleaf vs. WTleaf), of which 2137 genes were up-regulated and 1400 genes were down-regulated (Figure 8A,C). The top three up-regulated DEGs were *EXL1* (AT1G35140, log_2_FoldChange = 11.997), *TIFY* (AT2G34600, log_2_FoldChange = 9.602), and *ERF016* (AT5G21960, log_2_FoldChange = 9.362). The *EXL1* gene is involved in the C-starvation response. The *exl1* mutant showed diminished biomass production in a short-day/low light growth regime, impaired survival during extended night, and impaired survival under anoxic stress [45,46]. The top 3 down-regulated DEGs were *AT3G16670* (AT1G35140, log_2_FoldChange = −7.352), *WSD1* (AT5G37300, log_2_FoldChange = −6.296) and *CYP71B17* (AT3G26160, log_2_FoldChange = −6.259). *AT3G16670* belongs to the pollen Ole e 1 allergen and extensin family of proteins and is involved in the response to oxidative stress [47,48]. *WSD1* encodes a bifunctional enzyme, wax ester synthase (WS), and diacylglycerol acyltransferase (DGAT), which are involved in the triglyceride biosynthetic process and wax biosynthetic processes [49,50,51]. CYP71B17 encodes the *Arabidopsis* cytochrome P450 71B17 mRNA, which is involved in the oxidative degradation of various compounds and secondary metabolite biosynthetic processes [52,53].

The expression of 2429 genes was altered (|log_2_| ≥ 1, FDR ≤ 0.05) in the *pip1s*^−^ mutant siliques compared to the WT siliques (musili vs. WTsili), of which 1223 genes were up-regulated and 1206 genes were down-regulated (Figure 8A,D). The Venn diagram showed the overlap of 594 identical differentially expressed genes (DEGs) in the two comparison groups (muleaf vs. WTleaf and musili vs. WTsili) (Figure 8B). The top three up-regulated DEGs were AT3G06955 (AT3G06955, log2FoldChange = 8.777), *AT3G04180* (AT3G04180, log2FoldChange = 6.700) and *AT5G37690* (AT5G37690, log2FoldChange = 6.437). The top three down-regulated DEGs were *AIG1* (AT1G33960, log_2_FoldChange= −9.014), *CYP71B31* (AT3G53300, log_2_FoldChange= −7.133) and *AT2G15020* (AT2G15020, log_2_FoldChange= −7.055). Further analysis of DEGs found that many were related to photosynthesis, pollen wall formation, pollen development, pollen hydration and pollen tube growth (Table 1).

### 2.8. GO and KEGG Analyses of pip1s^−^ Mutants and WT plants

The gene Ontology (GO) analysis of DEGs in the group muleaf vs. WTleaf revealed 176 GO terms that were significantly altered at FDR < 0.05, including 130 biological processes, 22 cellular components and 24 molecular functions (Figure 5A). While in the group musili vs. WTsili, 162 terms with FDR < 0.05 were identified, including 112 biological processes, seven cellular components, and 43 molecular functions (Figure 5B). The top significantly altered terms were mainly related to metabolic processes, cellular processes, responses to stimulus and biological regulation, suggesting that the five *PIP1* genes played an important role in these processes.

**Figure 9:**
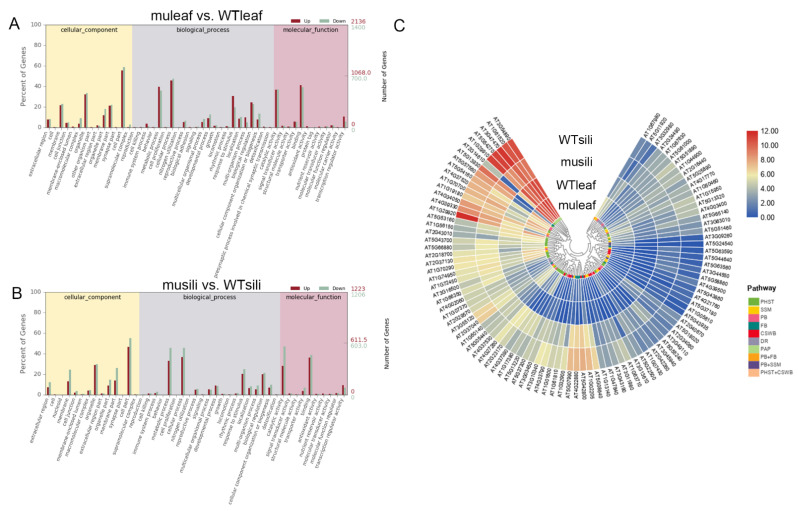
GO enrichment analysis and KEGG pathway analysis. (**A**,**B**) Gene Ontology (GO) analysis of cellular components, biological processes and molecular functions of muleaf vs. WT (**A**) and musili vs. WT (**B**). (**C**) KEGG pathway and cluster analysis of 99 DEGs. Plant hormone signal transduction (PHST); starch and sucrose metabolism (SSM); phenylpropanoid biosynthesis (PB); flavonoid biosynthesis (FB); cutin, suberin and wax biosynthesis (CSWB); DNA replication (DR); and photosynthesis (PAP).

In the Kyoto Encyclopedia of Genes and Genomes (KEGG) category, 881 transcripts were assigned to 117 pathways, of which 4 pathways were significantly enriched in group muleaf vs. WTleaf (*q* ≤ 0.05). Another 616 transcripts were assigned to 109 pathways, of which 7 pathways were significantly enriched in group musili vs. WTsili (Table 2). The KEGG pathway analysis showed that 47 hormone-related genes were significantly differentially expressed (including 26 up-regulated and 21 down-regulated) in the *pip1s*^−^ mutant rosette leaves compared to the WT rosette leaves. The analysis of secondary metabolism pathways using KEGG showed that the phenylpropanoids and flavonoid biosynthesis pathway were significantly altered in the rosette leaves and silique of the *pip1s*^−^ mutant compared to the WT plants. Some genes related to starch and sucrose metabolism and cutin, suberin and wax biosynthesis were significantly altered in the *pip1s*^−^ mutant silique compared to the WT siliques (Figure 9C).

### 2.9. Transcription Factor Analysis of the DEGs

A total of 150 and 69 transcription factor genes were significantly differentially expressed in the rosette leaves and siliques of the *pip1s^−^* mutant compared to the WT plant, respectively. Among the identified TF genes, many were involved in leaf, flower and seed development (Table 3 and Table 4). In rosette leaves, the TF genes related to leaf development covered many TF families, such as TCP, AP2, B3, MYB, NAC and WRKY. The expression of seven AP2 genes (ERF1A, ERF2, ERF6, ERF11, ERF018, ERF104, and ERF 109) were all up-regulated in the *pip1s*^−^ mutant compared to the WT plants, and the GO analysis indicated that these genes were involved in cell division [54,55]. Four NAC genes, including NAC59, NAC29, NAC42, and NAC84, involved in leaf senescence and cell division and expansion were all promoted in the *pip1s*^−^ mutant compared to the WT plants [56]. In the silique, the TF genes related to flower and seed development included the B-box domain proteins, zinc finger proteins, MYB, B3 and NAM TF families. The expression of four B-box domain protein genes (MIP1B, BBX32, AT5G54470, and AT4G27310) were all down-regulated in the *pip1s*^−^ mutant compared to the WT and the GO analysis indicated that these genes were involved in the regulation of flower development. Four zf-Dof genes, including *CDF1, CDF2, CDF3*, and *CDF5*, involved in flower development, were all repressed in the *pip1s*^−^ mutant compared to the WT plants.

### 2.10. Verification of Transcriptome Sequencing

**Figure 10:**
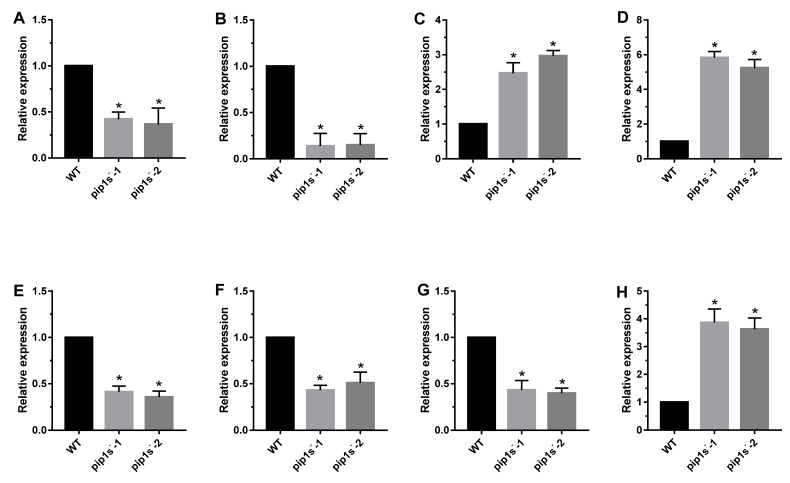
Q-PCR analysis of phenotype-related genes in the *pip1s^−^* mutant and WT plants. Relative expression of *LHCA1* (**A**), *LHCB1.1* (**B**), *RAV1* (**C**), *WRKY22* (**D**), *CALS7* (**E**), *CALS8* (**F**), *MYB103* (**G**) and *PMEI1* (**H**) in *pip1s^−^* mutant and WT. Bars represent means ± SD of three biological replicates. Significant differences were determined by one-way ANOVA followed by Duncan’s multiple range test (* *p* < 0.05).

Eight genes were selected for quantitative real-time PCR (Q-PCR) to confirm the reliability of the RNA-seq data (Figure 10). *LHCA1* and *LHCB1.1* encode chlorophyll a/b binding protein, as a light harvesting protein complex in photosystem (PS) I or II, which converts light energy into unstable chemical energy. *RAV1* was identified as a negative regulator of leaf development in *Arabidopsis*. *WRKY22* was reported to be related to leaf senescence and response to chitin. *CALS7* and *CALS8* are callose-synthesizing genes, and their deletion hinders the normal development of pollen outer walls. *MYB103* was reported to regulate the expression of *CALS7* and *CALS8*, and subsequently regulate the development of pollen tapetum. PMEI1 was identified as pectin methyl esterase inhibitor, which was located at the top of pollen tube cells and negatively regulated catalysis. *LHCA1*, *LHCB1.1*, *CALS7*, *CALS8* and *MYB103* were significantly down regulated, whereas *RAV1*, *WRKY22* and *PEMI1* were significantly up regulated in *pip1s^−^* mutant compared to the WT (Figure 10).

## 3. Discussion

### 3.1. Mutation of PIP1s Influences the Vegetative Growth of Arabidopsis

PIPs are usually located to the plasma membrane, but recent evidence suggests that some isoforms may also be located to the chloroplast envelope in at least minute amounts [57,58,59]. The co-localization of the PIPs and TIPs in chloroplast and thylakoid membranes may reflect their key roles in water supplying and CO_2_ transport for photosynthesis [60]. Some *PIP1s* appear to play a dual role in water and CO_2_ transport of *Arabidopsis* and tobacco [14,61,62,63]. The CO_2_ permeability of the chloroplast envelope purified from tobacco leaves is five times lower than that of plasma membrane vesicles. After antisense inhibition of *NtAQP1* in transgenic tobacco, its CO_2_ permeability is reduced by 90% [64]. In addition to *NtAQP1*, *AtPIP1;2* has also been shown to promote CO_2_ transmembrane transport after heterologous expression in yeast cells [65]. The genetic changes intheir functions in tobacco or *Arabidopsis* plants, using antisense suppression or overexpression, revealed a positive correlation between their expression and CO_2_ assimilation rate [65]. To date, the aquaporins that all belong to the subclasses of PIP1 and PIP2 have been shown to promote the membrane diffusion of CO_2_ in several plant species.

**Figure 11:**
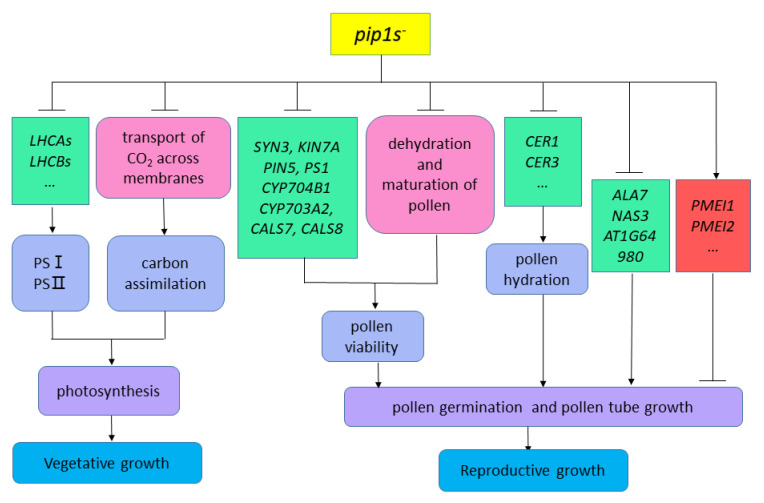
Hypothesis diagram of *pip1s^−^* mutants affecting plant vegetative and reproductive growth.

In our study, compared to the WT plants, the *pip1s^−^* mutants had smaller rosette leaf size and fewer rosette leaf numbers which exhibited overall defects in vegetative growth. The vegetative growth defect of the mutant may be caused by the mutation of *PIP1s* which limits the transportation of CO_2_ in plants, thereby affecting the second stage of photosynthesis in plants (Figure 11). On the other hand, seven down-regulated *LHCAs* and *LHCBs,* which encode the chlorophyll a/b binding protein, may affect the first stage of photosynthesis and block the conversion of light energy into unstable chemical energy (Figure 11). In addition, according to our previous studies, single *pip1^−^* mutants, double mutants, or even triple mutants had no significant effect on vegetative growth, implying that in addition to *AtPIP1;2*, other *PIP1s* genes are also involved in the active transport of CO_2_. The phenotype of plants with genetically altered aquaporins is usually difficult to decipher, because it integrates far more than the direct effects of aquaporins on tissue hydraulics or carbon fixation, but mutations in *PIP1s* may also regulate the expression of other AQPs or transcription factors and hormone signal transduction. According to our work, a hypothesis has been proposed that the mutation of *PIP1s* defect the vegetative growth of mutants by regulating the expression of chlorophyll a/b binding protein genes and the transmembrane transport of CO_2_ (Figure 11).

### 3.2. Mutation of PIP1s Influences the Reproductive Growth of Arabidopsis

Two desiccated forms of higher plant life, pollen and seeds, play an important role in the plant life cycle. Pollen and seeds express TIP5 and TIP3 specific aquaporin subclasses, respectively [66,67,68]. This specificity may result from the highly specialized growth or germination processes observed in pollen and seeds. In flowers, tissue desiccation involving aquaporins at various stages is required during reproductive growth. For example, dehydration of anthers is necessary for dehiscence and the release of mature pollen and is hindered by reduced expression of *PIP2s* in tobacco plants [69]. The maturation of pollen grains is accompanied by gradual dehydration, and their germination is induced by the rapid growth of pollen tubes, which also involves water transduction via PIPs. Previous research showed that after mutating two pollen TIPs specific to vegetative and sperm cells, *Arabidopsis* showed reduced fecundity in the presence of a limited water or nutrient supply [68].

According to our research, the number of viable pollen grains per anther in the *pip1s^−^* mutants was significantly less than the WT plants, potentially because the mutation of *PIP1s* affects the normal dehydration and maturation process of pollen grains. Additionally, the pollen from both the WT and the *pip1s^−^* mutants had regular honeycomb outer wall structures. However, pollen outer wall of the mutant was deformed and shrunken, which resulted in the impaired pollen-stigma binding ability of the *pip1s^−^* mutant compared to the WT plants. Additionally, the transcriptional sequence analysis showed that some key DEGs, including SYN3, KIN7A, PIN5, PS1, CYP704B1, CYP703A2, CALS7 and CALS8, were involved in exine formation and pollen development (Figure 11).

In addition, the pollen germination rate of the *pip1s^−^* mutant was significantly lower than the WT plants in vitro and in vivo. Mutations in *PIP1s* also regulate the expressions of many genes related to pollen tube growth and may block pollen grains from absorbing water and germinating (Figure 11).

The *pip1s^−^* mutants exhibited more undeveloped siliques, shorter siliques and fewer seeds. The lower numbers of siliques and seeds in the *pip1s^−^* mutant may be partly attributed to the withering of many flower buds of *pip1s^−^*before fertilization. Compared the developed siliques of the WT and *pip1s^−^* mutant plants, we found that the mutant siliques were nicked. In addition, compared to the WT plants, some aborted seeds and malformed seeds were observed in the *pip1s^−^* mutants. In summary, the change in pollen morphology and the reduced pollen viability may lead to a decrease in pollen adhesion and germination rate, that the siliques of the *pip1s^−^* mutants were shorter and nicked, finally leading to a significant decrease in the yield of the mutant.

The KEGG analysis showed that plant hormone signal transduction, phenylpropanoids and flavonoid biosynthesis, pentose and glucuronate interconversions, starch and sucrose metabolism and cutin, suberin and wax biosynthesis were significantly altered in *pip1s^−^* mutant siliques compared to WT siliques. The GO analysis of DEGs in the *pip1s^−^* mutant compared to the WT plants revealed that they were mainly related to metabolic processes, cellular processes, responses to stimulus and biological regulation, suggesting that *PIP1s* may be involved in regulating growth and development regulation and the stress response in plants. TF genes accounted for a large proportion of the DEGs identified in the *pip1s^−^* and consisted of many TF families, such as TCP, AP2, B3, MYB, NAC, NAM, WRKY, B-box domain proteins and zinc finger proteins. Most of the TF genes were related to cell proliferation, cell division, leaf development, leaf senescence, flower development, pollen development, pollen maturation, pollen sperm cell differentiation, pollen tube guidance, embryo sac development and embryo development ending in seed dormancy, suggesting that these TF genes play various important roles in plant growth and development [54,55,56].

## 4. Materials and Methods

### 4.1. Plant Materials and Growth Conditions

*Arabidopsis* ecotype Columbia (Col-0) was used as the WT control in the present study. The triple T-DNA insertion mutant used in this study was obtained through the hybridization method. Seeds were sterilized with 0.1% (*w/v*) HgCl_2_ for 10 min, washed five times with sterile water, sown on Murashige and Skoog (MS) medium [3% (*w/v*) sucrose, 0.7% (*w/v*) agar] and vernalized at 4 °C for 3 days in the dark. Then, 10-day-old seedlings were transferred to pots filled with a mixture of soil and sand (3:1) and grown in a chamber set at 22 °C; 110 μmol·m^−2^·s^−1^ light intensity; 16-h light/8-h dark cycle; and 70% relative humidity.

### 4.2. Generation of the pip1s^−^ Mutant

To generate the *pip1s^−^-1 and pip1s^−^-2* mutant, sgRNA targets in the *PIP1;4* and *PIP1;5* genes were selected and cloned into the pVK004-15 vector (Viewsolid biotech, Beijing, China), and then were transformed into the triple mutant by floral dip method. The transgenic T1 seeds were collected and screened on 1/2 medium containing 50 mg/L hygromycin. The fragments covering the mutation sites were amplified from the T1 transgenic lines by PCR and sequenced to identify the successfully mutated ones. The homozygous mutants were screened from the T2 generation and the seeds were harvested from individual lines to obtain T3 plants, of which non-hygromycin resistant plants were obtained.

### 4.3. Identification of the pip1s^−^ T-DNA Mutant

Homozygous T-DNA insertional mutant plants were confirmed by conducting two consecutive PCR assays. The first assay involved the use of two gene-specific primers: LP and RP. The second assay used one gene-specific primer, RP, and one T-DNA-specific primer (LB).

### 4.4. High-Throughput mRNA Sequencing Analysis

Total RNA was extracted from thirty-day-old rosette leaves and 14 DAF siliques and 3 μg of RNA from each sample were used for library construction and subsequent RNA-deep sequencing on the Illumina HiSeq 2500 platform. RNA-seq data were collected from two independent experiments. The adaptor sequences and low quality sequences were removed. Approximately 4.0 GB of clean reads were generated from each sample. The clean reads were mapped to the *Arabidopsis* reference genome using TopHat with TAIR10 gene annotation as the transcript index. The minimum and maximum intron lengths were set to 40 and 5000, respectively. Cufflinks was used to assemble the new transcripts. HTSeq was used to calculate the raw read counts for each gene. Gene expression was normalized among samples using DESeq. The differental gene expression data were collected from the comparison with a fold change ≥2 and a false discovery rate of 0.01.

### 4.5. RNA Extraction and qRT-PCR

Total RNA was extracted from leaves and siliques at different developmental stages using the RNeasy Plant Mini Kit (Qiagen, Amsterdam, The Netherlands). Total RNA (1 μg) from each sample was converted into cDNA by reverse transcription using the RNA PCR Kit (TaKaRa, Dalian, China) according to the manufacturer’s instructions. qRT-PCR was conducted on an ABI 7500 system (Applied Biosystems, New York, NY, USA) using the TransStart™ Green qRT-PCR SuperMix Kit (TransGen, Beijing, China). Actin2 was used as a reference gene to normalize the relative transcriptional abundance and to minimize differences in the copy numbers of cDNA templates. The control sample was conferred a value of 1. All data were calculated and analysed from three independent samples based on the 2^-ΔΔCt^ method. 

### 4.6. Statistical Analysis

The data are presented as means ± SD and were compared using SPSS software with one-way ANOVA followed by Duncan’s multiple range test at a significance level of *p* < 0.05.

## 5. Conclusions

The *pip1s^−^* quintuple mutant displayed severe growth defects in rosette leaves, flowers, siliques and seeds. Compared to the WT plant, the *pip1s^−^* mutants showed smaller and fewer rosette leaves, smaller flowers, shorter silique and fewer seeds under physiological conditions. Further studies on the pollens exhibited that pollen exine shape was abnormal and pollen vitality and germination rate are significantly reduced in the *pip1s^−^* mutants. In summary, *PIP1s* play a very important role in plant vegetative growth and reproductive growth and are important potential genetic resources in agronomic and crop science. Our research also provides a theoretical basis for agricultural production to improve crop traits and yield.

## Figures and Tables

**Table 1:** DEGs analysis related to growth and development of the *pip1s*^−^ mutant and the WT.

Gene ID	Gene Name	Description	Mutant Count	WT Count	Log_2_Fold Change	Padj
AT3G54890	*LHCA1*	Chlorophyll a/b binding protein, as a light harvesting protein complex in photosystem (PS) I or II, which converts light energy into unstable chemical energy.	15,708	39,004	−1.19727	0
AT1G61520	*LHCA3*		19,795	43,290	−1.01405	0
AT3G47470	*LHCA4*		22,052	66,725	−1.48247	0
AT1G29920	*LHCB1.1*		5349	82,295	−3.82861	0
AT2G05100	*LHCB2.1*		2706	46,267	−3.9809	0
AT5G54270	*LHCB3*		8926	51,223	−2.40585	0
AT3G08940	*LHCB4.2*		10,934	25,689	−1.11748	0
AT4G20050	*QRT3*	Encodes a polygalacturonase that plays a direct role in degrading the pollen mother cell wall.	159	570	−1.72708	8.15 × 10^−48^
AT1G69500	*CYP704B1*	Encodes a cytochrome P450, designated CYP704B1. Expressed in the developing anthers. Essential for pollen exine development.	84	193	−1.08529	4.44 × 10^−9^
AT1G01280	*CYP703A2*	Catalyzes the in-chain hydroxylation of medium-chain saturated fatty acids which is involved in pollen development.	78	181	−1.09959	9.40 × 10^−9^
AT1G06490	*CALS7*	Callose synthesis gene, its deletion causes the normal development of the outer wall of pollen to be blocked.	18	54	−1.55859	2.80 × 10^−5^
AT3G14570	*CALS8*		37	85	−1.17356	2.34 × 10^-5^
AT1G63910	*AtMYB103*	Regulate *CALS* expression to regulate pollen tapetum development and callose degradation.	46	107	−1.10305	9.13 × 10^−6^
AT3G59550	*SYN3*	Core components of meiotic and mitotic cohesin complexes, which plays an important role in pollen development.	11	26	−1.21464	0.012428
AT1G18370	*KIN7A*	Encodes a kinesin HINKEL and are required for cytokinesis in pollen.	4	36	−3.14355	1.72 × 10^−7^
AT5G16530	*PIN5*	Acts together with PIN8 in affecting pollen development and auxin homeostasis.	38	94	−1.28029	1.98 × 10^−6^
AT1G34355	*PS1*	Mutations in PS1 lead to diploid male spores, diploid pollen grains, and spontaneous triploid plants in the next generation.	3	19	−2.63659	0.000449
AT1G02205	*CER1*	Involved in the synthesis and transport of lipids in the oil-bearing layer, necessary for the signal transduction mechanism of pollen and stigma hydration.	4284	10,773	−1.21554	0
AT5G57800	*CER3*		5072	11,925	−1.11851	0
AT1G09240	*NAS3*	Encodes a nicotianamine synthase, which involved in pollen tube growth.	46	239	−2.26245	4.47 × 10^−29^
AT3G13900	*ALA7*	Encodes a P-type ATPases expressed in the pollen plasma membrane. Double mutants with ALA6 display pollen and pollen tube defects.	74	260	−1.69806	5.33 × 10^−22^
AT1G64980	*AT1G64980*	Encodes a putative nucleotide-diphospho-sugar transferase required for pollen germination and tube growth.	590	1476	−1.29653	7.03 × 10^−83^
AT3G62180	*AT3G62180*	Pectin methyl esterase inhibitor, located at the top of pollen tube cells, negatively regulates catalysis.	8	1	3.026373	0.011036
AT1G48020	*PMEI1*		18	5	1.87437	0.004514
AT3G17220	*PMEI2*		11	2	2.485805	0.007609

**Table 2:** KEGG pathway analysis of *pip1s*^−^ mutant RNA-sequencing data.

	Pathway	Up Count	Down Count	*q*-Value
muleaf vs. WTleaf	Plant hormone signal transduction	26	21	0.000195
	Phenylpropanoid biosynthesis	15	12	0.025966
	Flavonoid biosynthesis	6	3	0.016017
	DNA replication	0	12	0.019096
musili vs. WTsili	Phenylpropanoid biosynthesis	15	23	2.52 × 10^−7^
	Cutin, suberine and wax biosynthesis	6	12	0.000055
	Pentose and glucuronate interconversions	21	1	2.51 × 10^−5^
	Starch and sucrose metabolism	15	7	0.043843
	Circadian rhythm—plant	5	7	0.000399
	Photosynthesis—antenna proteins	0	7	0.003608
	Flavonoid biosynthesis	3	5	0.019055

**Table 3:** Analysis of TFs related to leaf development in the *pip1s*^−^ mutant and WT plants.

Gene ID	Gene Name	TF Family	GO	Muleaf Count	WTleaf Count	Log_2_Fold Change	Padj
AT1G69690	*TCP15*	TCP	GO:0008283|cell proliferation	117	284	−1.253	3.54 × 10^−16^
AT4G37750	*ANT*	AP2	GO:0042127|regulation of cell proliferation	35	83	−1.219	1.66 × 10^−5^
AT5G14960	*E2FD*	E2F/DP	GO:0008284|positive regulation of cell proliferation	57	26	1.159	0.000493
AT5G59340	*WOX2*	WOX	GO:0008284|positive regulation of cell proliferation	27	4	2.781	1.61 × 10^−5^
AT5G61600	*ERF104*	AP2	GO:0051301|cell division; GO:0045893|positive regulation of transcription	3324	338	3.324	0
AT5G47220	*ERF2*	AP2	GO:0051301|cell division; GO:0045893|positive regulation of transcription	2896	866	1.768	4.36 × 10^−259^
AT4G17500	*ERF1A*	AP2	GO:0051301|cell division	3616	317	3.538	0
AT4G17490	*ERF6*	AP2	GO:0051301|cell division	647	52	3.664	2.08 × 10^−129^
AT1G28370	*ERF11*	AP2	GO:0051301|cell division	1281	28	5.542	2.98 × 10^−304^
AT1G74930	*ERF018*	AP2	GO:0051301|cell division	3056	56	5.796	0
AT4G34410	*ERF109*	AP2	GO:0051301|cell division	58	0	6.884	1.43 × 10^−14^
AT5G06250		B3	GO:0048366|leaf development; GO:0045892|negative regulation of transcription	1	6	−2.559	0.036841
AT1G13260	*RAV1*	B3	GO:0048366|leaf development; GO:0045892|negative regulation of transcription	4965	2454	1.043	4.95 × 10^−199^
AT4G27950	*CRF4*	AP2	GO:0048366|leaf development	3	10	−1.711	0.036856
AT2G46310	*CRF5*	AP2	GO:0048366|leaf development	109	32	1.795	3.35 × 10^−11^
AT3G15030	*TCP4*	TCP	GO:0048366|leaf development; GO:0045962|positive regulation of development	498	1545	−1.607	3.59 × 10^−119^
AT5G48090	*ELP1*	MYB	GO:2000024|regulation of leaf development	8	1	3.026	0.011036
AT4G01250	*WRKY22*	WRKY	GO:0010150|leaf senescence	411	69	2.601	1.60 × 10^−60^
AT4G23810	*WRKY53*	WRKY	GO:0010150|leaf senescence; GO:0045893|positive regulation of transcription	991	488	1.048	5.10 × 10^−41^
AT3G29035	*NAC59*	NAC	GO:0010150|leaf senescence; GO:1900057|positive regulation of leaf senescence	894	410	1.151	5.56 × 10^−43^
AT1G69490	*NAC29*	NAC	GO:0010150|leaf senescence	718	291	1.329	2.84 × 10^−43^
AT2G43000	*NAC42*	NAC	GO:0010150|leaf senescence; GO:1900056|negative regulation of leaf senescence	118	43	1.483	1.83 × 10^−9^
AT5G14000	*NAC84*	NAC	GO:0010150|leaf senescence; GO:0045892|negative regulation of transcription	19	0	5.274	8.52 × 10^−6^
AT5G62165	*AGL42*	SRF-TF	GO:0010150|leaf senescence; GO:0045944|positive regulation of transcription	290	40	2.884	7.23 × 10^−48^

**Table 4:** Analysis of TFs related to flower and seed development in the *pip1s*^−^ mutant and WT plants.

Gene ID	Gene Name	TF Family	GO	Musili Count	WTsili Count	Log_2_Fold Change	Padj
AT5G51860	*AGL72*	SRF-TF	GO:0009908|flower development;	4	13	−1.586	0.027378
AT5G65080	*MAF5*	SRF-TF	GO:0009910|negative regulation of flower development	115	29	2.102	1.64 × 10^−^^14^
AT4G27310		zf-B_box	GO:0009909|regulation of flower development	146	1146	−2.858	5.04 × 10^−^^175^
AT5G54470		zf-B_box	GO:0009909|regulation of flower development	33	237	−2.729	2.07 × 10^−^^35^
AT3G21150	*BBX32*	zf-B_box	GO:0009909|regulation of flower development; GO:0045892|negative regulation of transcription	21	140	−2.622	1.35 × 10^−^^20^
AT3G21890	*MIP1B*	zf-B_box	GO:0009909|regulation of flower development	5	18	−1.733	0.007799
AT5G61380	*APRR1*	CCT	GO:0009908|flower development; GO:0010629|negative regulation of gene expression	4029	867	2.331	0
AT5G15845		CCT	GO:0009909|regulation of flower development	5	0	3.437	0.025562
AT5G41380		CCT	GO:0009909|regulation of flower development	14	5	1.600	0.018327
AT5G59990		CCT	GO:0009908|flower development; GO:0045892|negative regulation of transcription	59	26	1.297	9.15 × 10^−^^5^
AT3G02380	*COL2*	CCT	GO:0009909|regulation of flower development	54	455	−2.960	1.61 × 10^−^^72^
AT5G57660	*COL5*	CCT	GO:0009909|regulation of flower development	612	3534	−2.415	0
AT5G62430	*CDF1*	zf-Dof	GO:0009908|flower development; GO:0045892|negative regulation of transcription	80	397	−2.196	8.04 × 10^−^^46^
AT5G39660	*CDF2*	zf-Dof	GO:0009908|flower development	205	578	−1.381	2.87 × 10^−^^35^
AT3G47500	*CDF3*	zf-Dof	GO:0009908|flower development	66	847	−3.567	1.03 × 10^−^^157^
AT1G69570	*CDF5*	zf-Dof	GO:0009908|flower development	16	38	−1.133	0.005335
AT5G44190	*GLK2*	Myb_DNA-binding	GO:0009910|negative regulation of flower development	389	1220	−1.534	1.92 × 10^−^^85^
AT5G37260	*RVE2*	Myb_DNA-binding	GO:0009909|regulation of flower development	1178	175	2.866	2.81 × 10^-195^
AT2G34880	*JMJ15*	JmjC	GO:0009909|regulation of flower development; GO:0009555|pollen development; GO:0009793|embryo development ending in seed dormancy	9	21	−1.108	0.031931
AT3G20810	*JMJ30*	Cupin_8	GO:0009908|flower development	3870	61	6.102	0
AT4G01500	*NGA4*	B3	GO:0009908|flower development	87	200	−1.086	2.29 × 10^−^^9^
AT2G27300	*NTL8*	NAM	GO:0009908|flower development	18	6	1.700	0.006492
AT5G59570	*BOA*	UDPGT	GO:0009909|regulation of flower development	1055	112	3.351	4.67 × 10^−^^202^
AT2G45430	*AHL22*	DUF296	GO:0009908|flower development	79	174	−1.024	1.07 × 10^−^^7^
AT1G77950	*AGL67*	SRF-TF	GO:0009555|pollen development; GO:0010152|pollen maturation; GO:0080092|regulation of pollen tube growth	60	7	3.214	9.68 × 10^−^^13^
AT2G01200	*IAA32*	AUX_IAA	GO:0009555|pollen development; GO:0009793|embryo development ending in seed dormancy	63	2	5.092	6.00 × 10^−^^17^
AT1G02040		zf-C2H2_6	GO:0048235|pollen sperm cell differentiation	20	52	−1.264	0.000434
AT4G35610		zf-C2H2	GO:0048235|pollen sperm cell differentiation	20	10	1.115	0.039813
AT2G25230	*MYB100*	Myb_DNA-bind_6	GO:0010183|pollen tube guidance; GO:0009553|embryo sac development	10	2	2.437	0.011513
AT5G40360	*MYB115*	Myb_DNA-bind_6	GO:0010183|pollen tube guidance; GO:0009553|embryo sac development	19	47	−1.192	0.001401
AT1G65370		MATH	GO:0009553|embryo sac development	1289	651	1.100	6.01 × 10^−60^
AT1G59920		Filament	GO:0009793|embryo development ending in seed dormancy;	8	22	−1.345	0.016337
AT2G44735		DUF3308	GO:0009793|embryo development ending in seed dormancy	71	37	1.055	0.000212
AT4G01335		Gliadin	GO:0009793|embryo development ending in seed dormancy	13	0	4.815	0.000163

## Data Availability

The data are available upon reasonable request from qualified researchers.
